# Anatomical Shifts of the Bowel During Positioning: Relevance to Prone Lateral Access Surgery

**DOI:** 10.7759/cureus.102467

**Published:** 2026-01-28

**Authors:** Takashi Sono, Hiroshi Iwata, Yasuyuki Onishi, Takayosh Shimizu, Koichi Murata, Bungo Otsuki, Shuichi Matsuda

**Affiliations:** 1 Department of Orthopaedic Surgery, Kyoto University Graduate School of Medicine, Kyoto, JPN; 2 Department of Diagnostic Imaging and Nuclear Medicine, Kyoto University, Kyoto, JPN

**Keywords:** bowel displacement, lumbar lateral interbody fusion, prone position, supine position, vessel displacement

## Abstract

This radiographic analysis aimed to evaluate the differences in the anatomical position of the bowel relative to the lateral surgical corridor and the spine between prone and supine positions. Retroperitoneal transpsoas lateral lumbar interbody fusion can be performed in the prone position, allowing simultaneous lateral and posterior spinal access without repositioning; however, bowel positional changes associated with this approach have not been well characterized. From January 2020 to December 2024, 13 patients who underwent computed tomography (CT)-guided biopsy in the prone position with imaging spanning L2-L5 were retrospectively analyzed. Patient factors, including age, sex, body mass index, history of abdominal surgery, and malignancy, were recorded. Supine and prone CT scans obtained within one month were used to measure the distances from the posterior vertebral line to the bowel and abdominal aorta at the L2/3, L3/4, and L4/5 levels, and positional differences between postures were calculated. The cohort consisted of eight men and five women with a mean age of 60.2 years and a mean body mass index of 20.8; six patients had a history of abdominal surgery, and 11 had a history of cancer. Posterior bowel displacement was observed at all levels in the prone position, with mean changes of -0.6 mm at L2/3, -3.5 mm at L3/4, and -5.1 mm at L4/5, whereas the position of the abdominal aorta showed minimal change. Regression analysis identified female sex and prior abdominal surgery as significant factors which were statistically associated with bowel displacement at L3/4 and prior abdominal surgery and malignancy as significant factors at L4/5. Our data indicate consistent posterior bowel displacement in the prone position and suggest that female sex, malignancy, and prior abdominal surgery are associated with reduced bowel mobility, which has important implications for prone lateral lumbar surgical approaches.

## Introduction

Lumbar interbody fusion can be performed using several surgical approaches, each with distinct anatomical corridors, indications, and risk profiles. Posterior lumbar interbody fusion (PLIF) is performed through a midline posterior approach, allowing bilateral access to the disc space after retraction of the dural sac and nerve roots; although it provides direct decompression, it is associated with greater neural manipulation. Transforaminal lumbar interbody fusion (TLIF) is a modification of PLIF that uses a unilateral posterior approach through the intervertebral foramen, reducing dural retraction while maintaining the ability to achieve circumferential fusion. Anterior lumbar interbody fusion (ALIF) accesses the disc space through an anterior retroperitoneal or transperitoneal approach, enabling the placement of large interbody cages and the restoration of disc height and lumbar lordosis, but with potential risks to major vascular and visceral structures. Lumbar lateral interbody fusion (LLIF) is a well-established surgical approach for the management of lumbar degenerative disorders, providing substantial deformity correction and effective indirect decompression [1-3]. Since it was introduced in 2006, the number of LLIF surgeries has rapidly increased [1,4,5].

In LLIF, lateral cage insertion is performed with the patient in a lateral decubitus position, followed by repositioning to the prone position for pedicle screw placement from the posterior side. However, one major drawback is the time required for repositioning. To address this issue, single-position LLIF was developed. Since 2020, the prone transpsoas approach has been explored as a form of single-position LLIF [6-8].

Some studies have reported that LLIF in the prone position achieves a greater lumbar lordosis than LLIF performed in the lateral decubitus position [9,10]. However, few studies have evaluated how abdominal organs are displaced in the prone position relative to standard supine preoperative imaging. Therefore, this study aimed to investigate positional changes of the intestines and major blood vessels between the supine and prone positions, in order to provide clinically relevant information for planning and safely performing prone transpsoas spinal procedures.

## Materials and methods

Between January 2020 and December 2024, we identified 43 patients who underwent prone-position computed tomography (CT)-guided biopsy at Kyoto University Hospital, located in Kyoto, Japan.Of these patients, we selected 13 whose scans included the lumbar spine from L2 to L5. Patient background data, including age, sex, body mass index (BMI), prior abdominal surgery, and history of cancer, were evaluated as factors that could potentially affect abdominal organ displacement.

**Figure 1:**
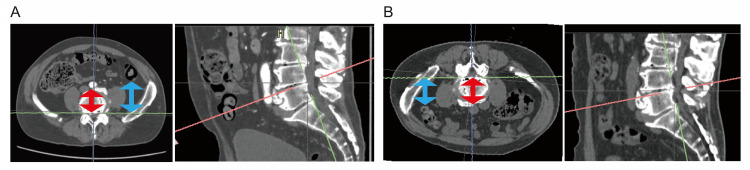
CT images of the lumbar spine in supine and prone positions (A) Left: CT image perpendicular to the L4/5 intervertebral disc space in supine position. Right: Sagittal reconstruction image. Blue arrows indicate the distance of the bowel at the left side to the posterior vertebral line. Red arrows indicate the distance of the abdominal aorta to the posterior vertebral line. (B) Left: CT image perpendicular to the L4/5 intervertebral disc space in prone position. Right: Sagittal reconstruction image. Blue arrows indicate the distance of the bowel at the left side to the posterior vertebral line. Red arrows indicate the distance of the abdominal aorta to the posterior vertebral line. CT: computed tomography

We measured distance from the posterior vertebral line to the bowel (distance of the bowel to the posterior vertebral line (DBV)) and distance to the abdominal aorta (distance of the abdominal aorta to the posterior vertebral line (DAV)) from CT scans obtained in both supine and prone positions taken within one month (Figure 1). The changes in each measurement (ΔDBV=DBV prone-DBV supine; ΔDAV=DAV prone-DAV supine) were assessed at the intervertebral disc levels from L2/3 to L4/5.

The CT scans were acquired using multidetector scanners. Image processing was performed using Aquarius Net Viewer (TeraRecon, Durham, North Carolina, United States). Prone CT imaging was obtained with bolsters placed under the chest, with both arms positioned forward and the hips extended. Measurements were performed three times on separate occasions by two surgeons (T.S. and H.I.), who were not blinded to patient position. The final value for each parameter was calculated as the mean of these measurements. Interobserver reliability, assessed using the intraclass correlation coefficient (ICC (2,1)), was 0.98 for DBV and 0.84 for DAV. Intraobserver reliability, assessed using ICC (1,3), was 0.98 for DBV and 0.90 for DAV. All ICC analyses were performed using Microsoft Excel (Version 2021; Microsoft, Redmond, Washington, United States). Stepwise multiple regression analyses were conducted using JMP Pro (Version 13.0; SAS Institute Inc., Cary, North Carolina, United States). All candidate variables entered into the regression model included age, sex, BMI, history of abdominal surgery, and history of malignancy. Stepwise selection was performed using a p-value threshold of 0.20, with both forward and backward variable entry and removal allowed and variable combinations evaluated according to this criterion. Multicollinearity was assessed by examining variance inflation factors, and no clinically relevant multicollinearity was identified among the included variables. Statistical significance was set at p<0.05.

This study was approved by the Kyoto University Graduate School of Medicine, Faculty of Medicine, and Hospital Medical Ethics Committee (approval number: R5161) and was conducted in compliance with the principles of the Declaration of Helsinki. The requirement for informed consent was waived due to the retrospective design of the study.

## Results

The study included 13 patients (eight males and five females) with a mean age of 60.2 years. The average BMI was 20.8. Six patients had a history of abdominal surgery, and 11 had a history of cancer (Table 1).

**Table 1:** Patient demographics

	Mean±SD
Age	60.2±18.2
Sex (male/female)	8/5
Body mass index	20.8±2.2
Malignancy (present/absent)	11/2
Abdominal operation (present/absent)	6/7

DBV in the prone position was measured to be 30.9 mm at L2/3, 23.0 mm at L3/4, and 20.4 mm at L4/5. DBV in the supine position was measured to be 31.5 mm at L2/3, 26.5 mm at L3/4, and 25.5 mm at L4/5. DAV in the prone position was measured to be 44.9 mm at L2/3, 45.1 mm at L3/4, and 44.5 mm at L4/5. DAV in the supine position was measured to be 45.3 mm at L2/3, 47.1 mm at L3/4, and 44.2 mm at L4/5.

ΔDBV was -0.6 mm at L2/3, -3.5 mm at L3/4, and -5.1 mm at L4/5. ΔDAV was -0.4 mm at L2/3, -1.9 mm at L3/4, and 0.3 mm at L4/5 (Table 2).

**Table 2:** DBV and DAV in the prone and supine positions The values in the table are presented as mean±standard deviation. DBV: the distance of the bowel to the posterior vertebral line; DAV: the distance of the abdominal aorta to the posterior vertebral line; ΔDBV: the difference between the distance of the bowel to the posterior vertebral lines in prone and supine positions; ΔDAV: the difference between the distance of the abdominal aorta to the posterior vertebral lines in prone and supine positions

Levels	DBV prone	DBV supine	ΔDBV	DAV prone	DAV supine	ΔDAV
L2/3	30.9±24.3	31.5±34.7	-0.6±18.6	44.9±9.0	45.3±8.6	-0.4±3.1
L3/4	23.0±21.5	26.5±30.4	-3.5±15.7	45.1±7.6	47.1±8.4	-1.9±6.0
L4/5	20.4±18.4	25.5±19.5	-5.1±8.2	44.5±7.7	44.2±9.1	0.3±3.3

ΔDBV showed negative values at all levels, indicating posterior displacement of the bowel in the prone position. In contrast, ΔDAV showed minimal change across all levels. Independent significant contributors for ΔDBV at L3/4 were identified as history of abdominal surgeries and being female. Independent significant contributors for ΔDBV at L4/5 were identified as history of cancer and abdominal surgeries (Table 3).

**Table 3:** Factors affecting ΔDBV in L3/4 and L4/5 identified using multiple regression analysis *: p<0.05 ΔDBV: the difference between the distance of the bowel to the posterior vertebral lines in prone and supine positions

	Factors	R^2^	Standard β	P-value
ΔDBV in L3/4	Abdominal operation	0.61	0.61	0.04*
	Female		0.66	0.03*
ΔDBV in L4/5	Abdominal operation	0.67	0.64	0.01*
	Malignancy		0.62	0.01*

## Discussion

This study investigated bowel and vascular displacement in the prone position, particularly the differences across lumbar intervertebral levels. The results demonstrated that at all lumbar intervertebral disc levels, the bowel tended to shift posteriorly in the prone position. This observation was contrary to our expectations and the previous report [11]. This unexpected finding suggested that prone positioning may not uniformly promote anterior bowel displacement across all lumbar levels, raising concerns about the generalizability of surgical planning based on assumptions at a single level.

Another significant finding was that the positioning of the major abdominal vessels, such as the aorta, remained relatively stable across different body positions, which corresponded to the previous report [12]. This implies that vascular displacement is minimal and might not be significant in altering the surgical access routes during prone transplant procedures.

Multivariate analysis identified three key patient-related factors that were associated with reduced bowel mobility: female sex, history of cancer, and abdominal surgery. Male patients may experience elevated intra-abdominal pressure that cannot be sufficiently relieved in the prone position, thereby promoting posterior bowel movements. Although previous studies by Kim et al. [13] have reported no significant associations between BMI and movement of abdominal organs, these studies focused on solid organs such as the liver and kidneys rather than the more mobile intestinal tract. Thus, the current study highlights the importance of specifically examining bowel behavior, which may respond differently to positional and pressure changes, in order to mitigate the risk of bowel injury during surgery, which has been reported to have a mortality rate of 13% [14].

Additionally, a prior history of cancer, especially in patients who undergo abdominal radiotherapy or surgical interventions, may affect the flexibility and positioning of the abdominal organs. Radiation-induced fibrosis or postoperative adhesions can anchor portions of the bowel, restricting natural displacement during postural changes. This is supported by the findings of Ganeshan et al., who emphasize the lasting effects of cancer therapies on organ mobility and spatial anatomy [15].

This study has several limitations. First, the small sample size and highly selected cohort of oncologic patients undergoing CT-guided biopsy reduced the statistical power and generalizability of our findings. Second, the absence of a four‑point support system may have had a larger effect on organ position than anticipated, potentially contributing to the observed posterior displacement. Third, variations in the positioning of the bowels and blood vessels due to differences in the respiratory cycle and the fact that prone and supine CT scans were performed on different days were not taken into account.

## Conclusions

Although the prone transpsoas approach may offer advantages in lumbar spinal surgery, surgeons should exercise caution, particularly in patients with a history of cancer or abdominal surgery. These populations may have altered intra-abdominal dynamics, which increases the risk of bowel injury. Preoperative imaging in both the supine and prone positions may be considered in selected high-risk patients to optimize surgical planning and safety.
